# Efficacy and Safety of *Brucea javanica* Oil Emulsion Injection in the Treatment of Gastric Cancer: A Systematic Review and Meta-Analysis

**DOI:** 10.3389/fnut.2021.784164

**Published:** 2021-12-09

**Authors:** Xinmiao Wang, Heping Wang, Luchang Cao, Jingyuan Wu, Taicheng Lu, Shixin Li, Jie Li

**Affiliations:** ^1^Department of Oncology, Guang'anmen Hospital, China Academy of Chinese Medical Sciences, Beijing, China; ^2^Graduate College, Beijing University of Traditional Chinese Medicine, Beijing, China

**Keywords:** *Brucea javanica* oil emulsion injection, gastric cancer, efficacy, safety, meta-analysis

## Abstract

**Background:** Gastric cancer (GC) is one of the most common digestive tract cancers and ranks fifth in the incidence of malignant tumors worldwide. *Brucea javanica* oil emulsion injection (BJOEI), a Chinese patent medicine extracted from *Brucea javanica* (Yadanzi in Chinese Pinyin), is widely used as an adjuvant treatment for GC in China. This systematic review and meta-analysis aimed to evaluate the available data on the efficacy and safety of BJOEI in the treatment of GC and assess the quality of the synthesized evidence.

**Methods:** A comprehensive search was performed on PubMed, EMBASE, CENTRAL, Web of Science, Chinese Biomedical Literature Database (CBM), China National Knowledge Infrastructure (CNKI), Wanfang database and Chinese Scientific Journals Database (VIP database), and other potential resources, such as the Chinese Clinical Trial Registry (ChiCTR) and ClinicalTrials.gov from their inception to July 31, 2021. Randomized controlled trials (RCTs) comparing the therapeutic effects of BJOEI combined with conventional therapy to those of conventional therapy alone were included. We used RevMan 5.3 for data analysis and quality evaluation of the included studies and assessed the evidence quality based on the Grading of Recommendations Assessment, Development, and Evaluation (GRADE) criteria.

**Results:** Eighteen RCTs involving 1,210 patients were included, and the meta-analysis results demonstrated that compared with the control group (conventional therapy), the experimental group (BJOEI combined with conventional therapy) showed a significantly improved overall response rate (ORR) (risk ratio [RR] = 1.52, 95% CI: 1.36–1.69, *P* < 0.00001), clinical benefit rate (CBR) (RR = 1.17, 95% CI: 1.11–1.23, *P* < 0.00001), performance status (RR = 1.72, 95% CI: 1.46–2.01, *P* < 0.00001), and reduced incidence of the following adverse drug reactions (ADRs): neutropenia, leukopenia, nausea and vomiting, diarrhea, liver damage, hand-foot syndrome, and peripheral sensory nerve toxicity. Subgroup analysis showed that the BJOEI intervention could significantly improve the ORR and CBR in patients with GC when combined with FOLFOX4, XELOX, and other chemotherapeutics.

**Conclusion:** The evidence presented in this study supports the fact that BJOEI combined with conventional chemotherapy provides a statistically significant and clinically important effect in the improvement of ORR, CBR, performance status, and ADR reduction in patients with GC. To further support this conclusion, more rigorously designed, large-scale, and multicenter RCTs are needed in the future.

## Introduction

Gastric cancer (GC) is one of the most common malignant tumors of the digestive tract and ranks fifth in incidence worldwide (1). There were ~1.089 million new cases of GC worldwide, of which 43.9% were reported in China, in 2020 (2). Due to the lack of specific symptoms in early GC, the diagnosis is often made at an advanced disease stage, and the mortality rate is high (3). At present, radical resection is still the main GC treatment, but most patients experience recurrence within 3 years after surgery. The postoperative recurrence rate of patients with locally advanced GC is as high as 50–80%. Once patients experience recurrence and metastasis after the operation, even if palliative chemotherapy is administered again, the 5-year survival rate remains low (4–7). Moreover, molecularly targeted therapy and immunotherapy of GC lag behind those of many other tumor types, and better survival benefits are still being explored (8).

Traditional Chinese Medicine (TCM) has a long historical tradition and currently attracts extensive attention because of its potential treatment benefits in the field of oncology. Our team has been committed to investigating the preventive and therapeutic values of TCM for many years (9–11). *Brucea javanica* oil emulsion injection (BJOEI) is a Chinese patent medicine extracted from *Brucea javanica* (*Yadanzi* in Chinese *Pinyin*). Its main active component is quassinoid sand fatty acids, which exert anticancer effects through multiple mechanisms (12). Studies have shown the synergistic effects of BJOEI combined with chemoradiotherapy on tumor attenuation, such as reversal of chemotherapy resistance, reduction of the recurrence and metastasis rates, and improvement of the quality of life (13–16). Although several existing systematic reviews have been conducted to evaluate the clinical efficacy of BJOEI in GC, none of them assessed the quality of the synthesized evidence and arrived at definitive conclusions (13, 17–19). The most recent one was reported by Wu et al. in 2018, in which the retrieval deadline was January 2017 (17). With the growing number of studies on the value of BJOEI in GC treatment, more randomized controlled trials (RCTs) have been published in recent years (20–23). Therefore, we conducted a systematic review to evaluate all available evidence of the efficacy and safety of BJOEI in the treatment of GC and assessed the quality of the synthesized evidence.

## Methods

This study was conducted according to the Preferred Reporting Items for Systematic Reviews and Meta-analyses (PRISMA) reporting guidelines, and readers can access the protocol of this systematic review in the International Prospective Register of Systematic Reviews (CRD42021265646).

### Inclusion Criteria

Studies that met the following criteria were included: (1) the study design was limited to RCTs, whether it was blinding or not; (2) the studies needed to meet the diagnostic criteria for GC by biopsy or postoperative pathological examination; and (3) studies provided the experimental group with BJOEI in combination with the same interventions provided to the control group.

### Exclusion Criteria

Studies were excluded if any of the following reasons were involved: (1) duplicate studies; (2) inappropriate interventions; (3) incomplete data; and (4) irrelevance to outcome indicators.

### Outcome Measures

Primary outcome measures included the overall response rate (ORR) and clinical benefit rate (CBR). The secondary outcome measure was the performance status. Safety outcome measures included the occurrence of adverse drug reactions (ADRs).

### Literature Search Strategy

We searched the following relevant databases from inception to July 31, 2021: PubMed, EMBASE, CENTRAL, Web of Science, the Chinese Biomedical Literature Database (CBM), the China National Knowledge Infrastructure (CNKI), Wanfang database, and Chinese Scientific Journals Database (VIP database), and other potential resources, such as the Chinese Clinical Trial Registry (ChiCTR) and ClinicalTrials.gov for more study records. The combination of MeSH terms and text words was applied to study retrieval. “Stomach Neoplasms” was regarded as the MeSH term. All the strategies were adapted from different databases. The search strategies used in PubMed were as follows:

#1 “Stomach Neoplasms” [MeSH]
#2 “Stomach Neoplasms*” [Title/Abstract] OR “Gastric Cancer*” [Title/Abstract] OR “Gastric Carcinoma” [Title/Abstract] OR “Gastric Neoplasm*” [Title/Abstract] OR “Cancer of Stomach” [Title/Abstract] OR “Stomach Cancer*” [Title/Abstract]
#3 #1 OR #2
#4 “Javanica oil emulsion injection” [Title/Abstract] OR “Yadanzi” [Title/Abstract] OR “Brucea javanica oil emulsion” [Title/Abstract] OR “Brucea javanica” [Title/Abstract]
#5 #3 AND #4

### Study Selection

The search results were imported into Excel 2003. After removing duplicates, the titles and abstracts were screened for potential studies. Then, the full articles were checked to determine whether the studies met the inclusion criteria. The study selection process was independently performed by two investigators.

### Data Extraction and Quality Assessment

All data were independently extracted by two investigators, and any discrepancies between the reviewers were resolved by the intercessor (JL) until consensus was reached. Data retrieved from the publications included author name, year of publication, number of patients, average age, gender, details about dosage and course of treatment, and outcome data. When necessary and feasible, the corresponding authors of the selected studies were contacted to obtain missing or incomplete data.

In terms of bias, the articles were evaluated as low risk, high risk, and unclear risk according to the following quality items: randomization generation, allocation concealment, subject blinding, outcome assessment, incomplete outcome data, and selective outcome reporting.

### Statistical Analysis

Quantitative synthesis was conducted for outcomes reported in more than one homogeneous RCT. The systematic review was performed using the RevMan 5.3 software. Random-effects or fixed-effects models were chosen based on the analysis of heterogeneity. Randomized individuals were considered as unit-of-analysis issues. If a meta-analysis was not appropriate because of clinical/methodological issues or statistical heterogeneity, a narrative summary of the findings or relevant subgroup analyses were used. The RR was used to evaluate dichotomous outcomes, while the mean difference (MD) was used to assess continuous variables. Each outcome numerical value was presented with 95% CIs. Funnel plots were used to test the risk of publication bias. The heterogeneity between RCTs was analyzed using the chi-square test and estimated using *I*^2^. Results of *P* ≥ 0.1 and *I*^2^ ≤ 50% suggested a lack of significant heterogeneity, and a fixed-effects model was used accordingly; otherwise, the random-effects model was used. When conducting the meta-analysis, several subgroup analyses were performed to identify subpopulations that might be associated with differences in efficacy. The results of the sensitivity analysis were reported.

### Quality of the Synthesized Evidence

Quality assessment of the synthesized evidence was performed using the Grading of Recommendations Assessment, Development, and Evaluation (GRADE) approach (24). This assessment of evidence quality includes the risk of bias, heterogeneity, indirectness, imprecision, and publication bias. The quality of the evidence was classified as high, moderate, low, or very low.

## Results

### Literature Search Results

**Figure 1:**
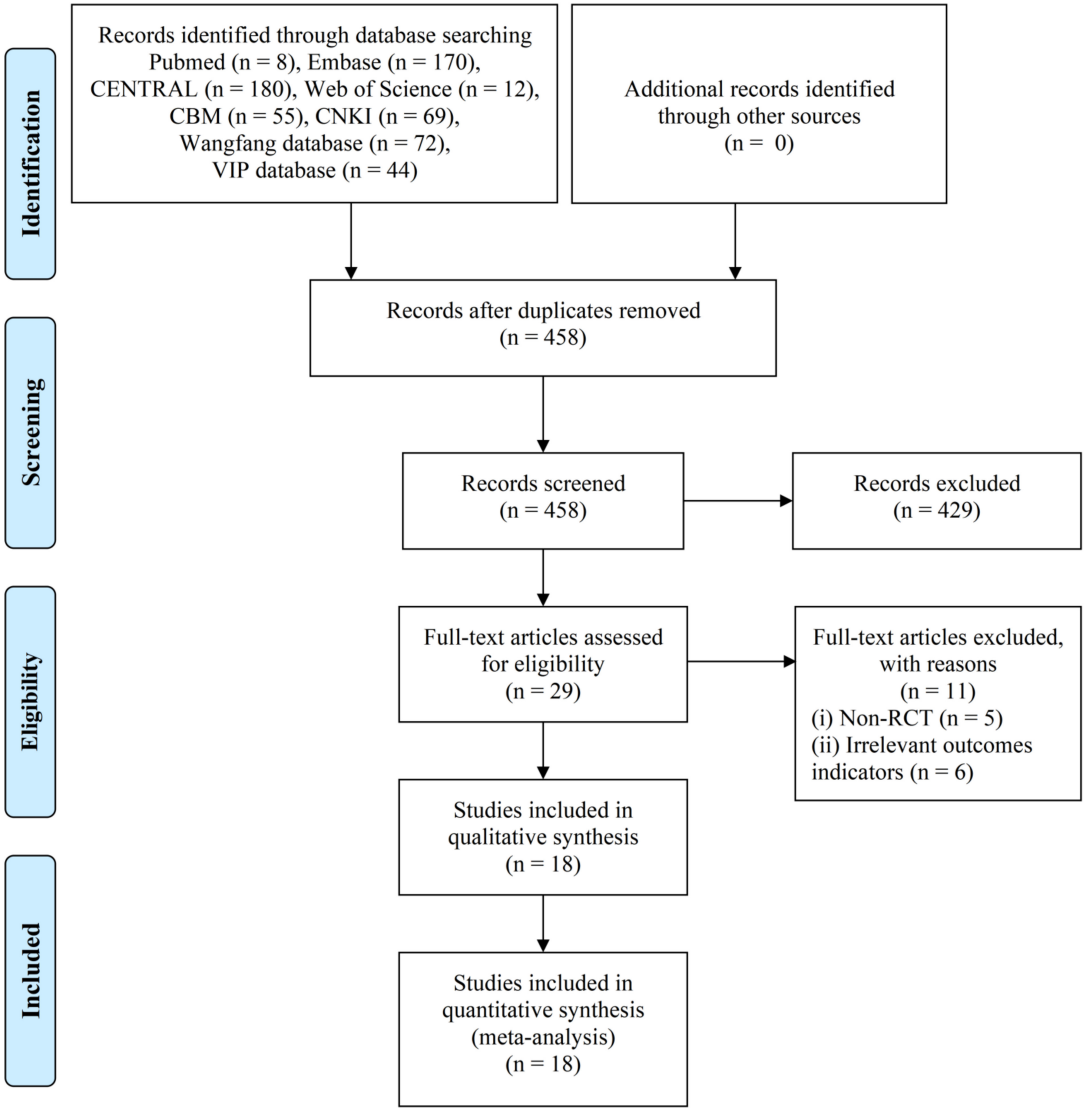
PRISMA flow diagram of the literature search process. PRISMA, the Preferred Reporting Items for Systematic Reviews and Meta-Analysis.

A total of 458 clinical studies were identified based on the retrieval strategy. After screening based on the inclusion/exclusion criteria, 18 articles were selected for further analysis (Figure 1).

### Study Description

Eighteen RCTs (20–23, 25–38) were included in this study, involving 1,210 patients with 618 cases in the experimental group and 592 cases in the control group. Furthermore, a total of four RCTs (20, 34, 36, 37) adopted BJOEI + FOLFOX4, and four RCTs (28, 29, 31, 38) employed BJOEI + XELOX. Due to the diverse combination therapy of BJOEI, subgroup analysis was considered. Additional details are summarized in Table 1.

**Table 1:** The characteristics of the included trials.

**References**	**No**	**Gender (M/F)**		**Age (year)**		**Interventions**		**Course (week)**	**Outcomes**
	**T/C**	**T**	**C**	**T**	**C**	**T**	**C**	**T/C**	
Cui (20)	60/60	40/20	36/24	51.43 ± 9.86	50.76 ± 10.63	BJOEI 30 ml+ FOLFOX4	FOLFOX4	4/4	①②③
Deng et al. (25)	21/21	29/13		39–81 (mean 60.2)		BJOEI 30 ml + DDP+MMC+VP-16	DDP+MMC+VP-16	–	①②③
Fan et al. (26)	24/18	14/10	13/5	70–85	70–85	BJOEI 30 ml + mFOLFOX4	mFOLFOX4	12/12	①②③
Gao (27)	26/26	14/12	15/11	32–79	35–75	BJOEI 30 ml+ MC/CF	MC/CF	4/4	①③
Jiang et al. (28)	32/32	21/11	20/12	36–64	32/63	BJOEI 30 ml+XELOX	XELOX	6/6	①②③
Li et al. (29)	40/40	22/18	21/19	64.5 ± 4.1	63.7 ± 3.4	BJOEI 30–50 ml+ XELOX	XELOX	12/12	①②③
Liu et al. (30)	40/38	30/10	26/12	29–71	34–68	BJOEI 30 ml + DX	DX	6/6	①②③
Ma et al. (31)	58/50	46/12	42/8	46.52 ± 5.13	47.13± 5.42	BJOEI 20 ml + XELOX	XELOX	12/12	①②③
Tan and Zhang (21)	20/20	11/9	12/8	51.53 ± 2.98	53.42 ± 3.22	BJOEI 20 ml+ DP	DP	6/6	②③
Tong and Hu (22)	42/42	30/12	28/14	54.69 ±8.42	54.41 ± 8.25	BJOEI 30 ml + SOX	L-OHP+TS-1	6/6	①③
Wang et al. (33)	31/31	17/14	16/15	29–63 (mean 50.2)		BJOEI 30 ml+ XELOPAC	XELOPAC	12/12	①③
Wang and Yang (34)	24/23	13/11	13/10	31–75	32–74	BJOEI 30 ml+ FOLFOX4	FOLFOX4	8/8	①②③
Wang (35)	38/30	23/15	19/11	32–71	35/69	BJOEI 30 ml + 5-FU+HCPT+CF+RT	5-FU+HCPT+CF+RT	9–12.86/9–12	①②③
Wang (36)	31/29	20/11	20/9	52.3 ± 12.71	51.6 ± 12.39	BJOEI 30 ml+ FOLFOX4	FOLFOX4	12.86/12	①②③
Wu et al. (37)	50/50	38/12	33/17	34–78	31–82	BJOEI 30 ml + FOLFOX4	FOLFOX4	4/4	①③
You et al. (23)	19/23	15/4	14/9	28–75	36–71	BJOEI 20–40 ml + TX	TX	6/6	①③
Zhang et al. (38)	41/41	28/13	26/15	68.8 ± 3.8	68.6 ± 5.2	BJOEI 30 ml + XELOX	XELOX	9/9	①②③
Wang et al. (32)	22/21	25/18		70–85		BJOEI 30 ml + UFT+FA	UFT+FA	16.57–24.86/16.57–24.86	①②③

*No, number of participants; T, treatment; C, control; M, male; F, female; Y, year; W, week; BJOEI, Brucea javanica Oil Emulsion injection; FOLFOX4, 5-FU+L-OHP+CF/THFA; mFOLFOX, 5-FU+L-OHP+CF; MC/CF, MMC+CF+5-FU; XELOX, L-OHP+CAP; DX, DXT+CAP; DP, DXT+DDP; SOX, L-OHP+TS-1; XELOPAC, PTX+CAP; TX, PTX/DXT+CAP; 5-FU, 5-Fluorouracil; L-OHP, Oxaliplatin; CF, Calcium Folinate; DDP, Cisplatin; MMC, Mitomycin-C; VP-16, Etoposide; CAP, Capecitabine; DTX, Docetaxel; TS-1, Tegafur; PTX, Paclitaxel; HCPT, Hydroxycamptothecin; RT, Radiotherapy; THFA, Tetrahydrogen folic acid; UFT, Tegafur-Uracil; FA, Folic acid. (1) Clinical total effective rate; (2) performance status; (3) adverse drug reactions; (4) adverse events; (5) withdrawals for any reason*.

### Quality Evaluation of the Literature

**Figure 2:**
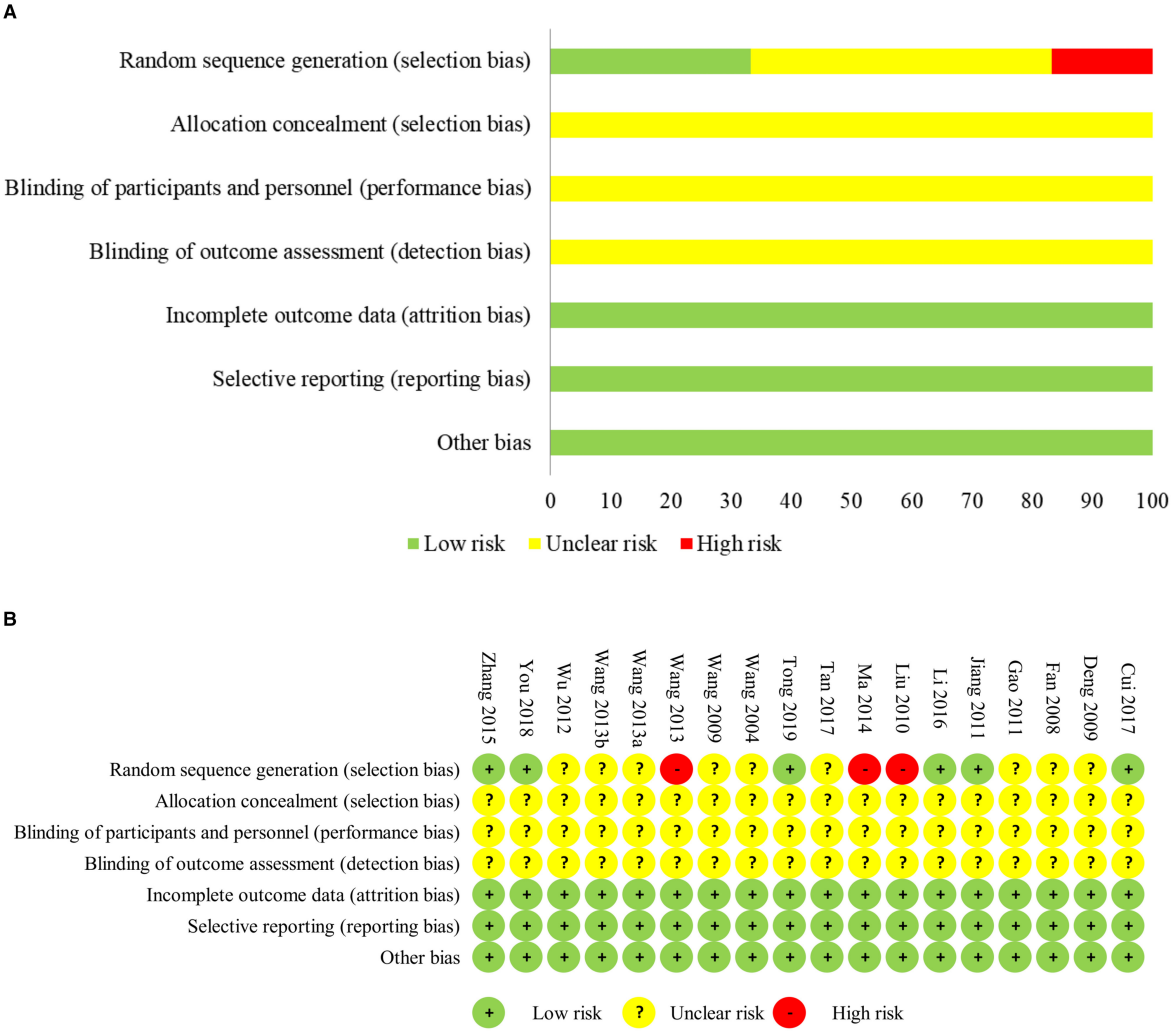
Risk of bias of included study. **(A)** Risk of bias summary, **(B)** risk of bias graph.

As shown in Figure 2, in terms of random sequence generation, six RCTs (20, 22, 23, 28, 29, 38) were considered to have a low bias risk by applying a random number table or random envelope. Three RCTs (30, 31, 36) were marked as “high risk” because they divided patients according to hospitalization period, ID, and postoperative chemotherapy, respectively. The other nine RCTs (21, 25–27, 32–35, 37) did not describe the specific randomized method and were evaluated as “uncertain risk.” None of the trials reported the methods of allocation concealment and blinding procedures, which indicated that there were unclear bias risks.

### Outcome Measures

#### Primary Outcomes

##### ORR

**Figure 3:**
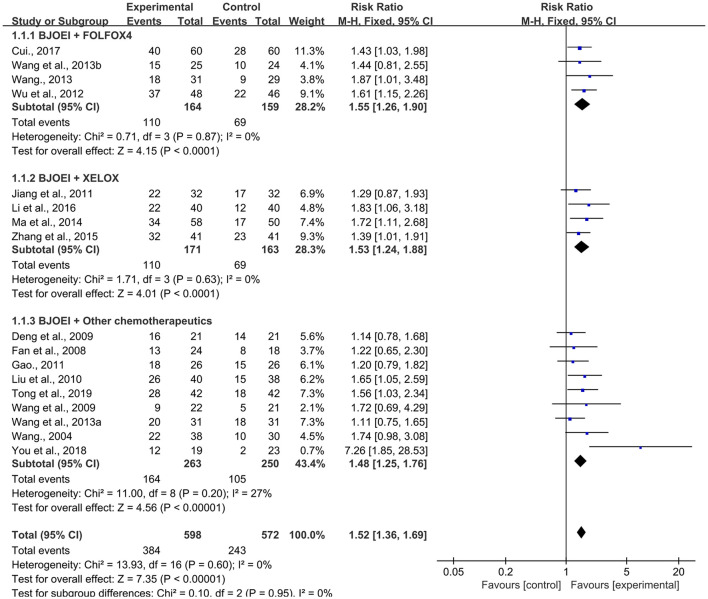
Forest plot of improvement of overall response rate.

In total, 17 RCTs (20, 22, 23, 25–38) with 1,170 patients presented ORR data. To explore the potential effect differences in ORR, we conducted a subgroup analysis according to the different combination therapies of BJOEI, namely, BJOEI + FOLFOX4, BJOEI + XELOX, and BJOEI + other chemotherapeutics. As shown in Figure 3, the results demonstrated that compared with the control group, the experimental group of patients with GC exhibited a significantly improved ORR (RR = 1.52, 95% CI: 1.36–1.69, Z = 7.35, *P* < 0.00001). Furthermore, subgroup analysis showed that there were statistically significant differences in ORR between the BJOEI intervention and control groups in patients who received BJOEI combined with FOLFOX4 (RR = 1.55, 95% CI: 1.26–1.90, Z = 4.15, *P* < 0.0001), XELOX (RR = 1.53, 95% CI: 1.24–1.88, Z = 4.01, *P* < 0.0001), and other chemotherapeutics (RR = 1.48, 95% CI: 1.25–1.76, Z = 4.56, *P* < 0.00001).

##### CBR

**Figure 4:**
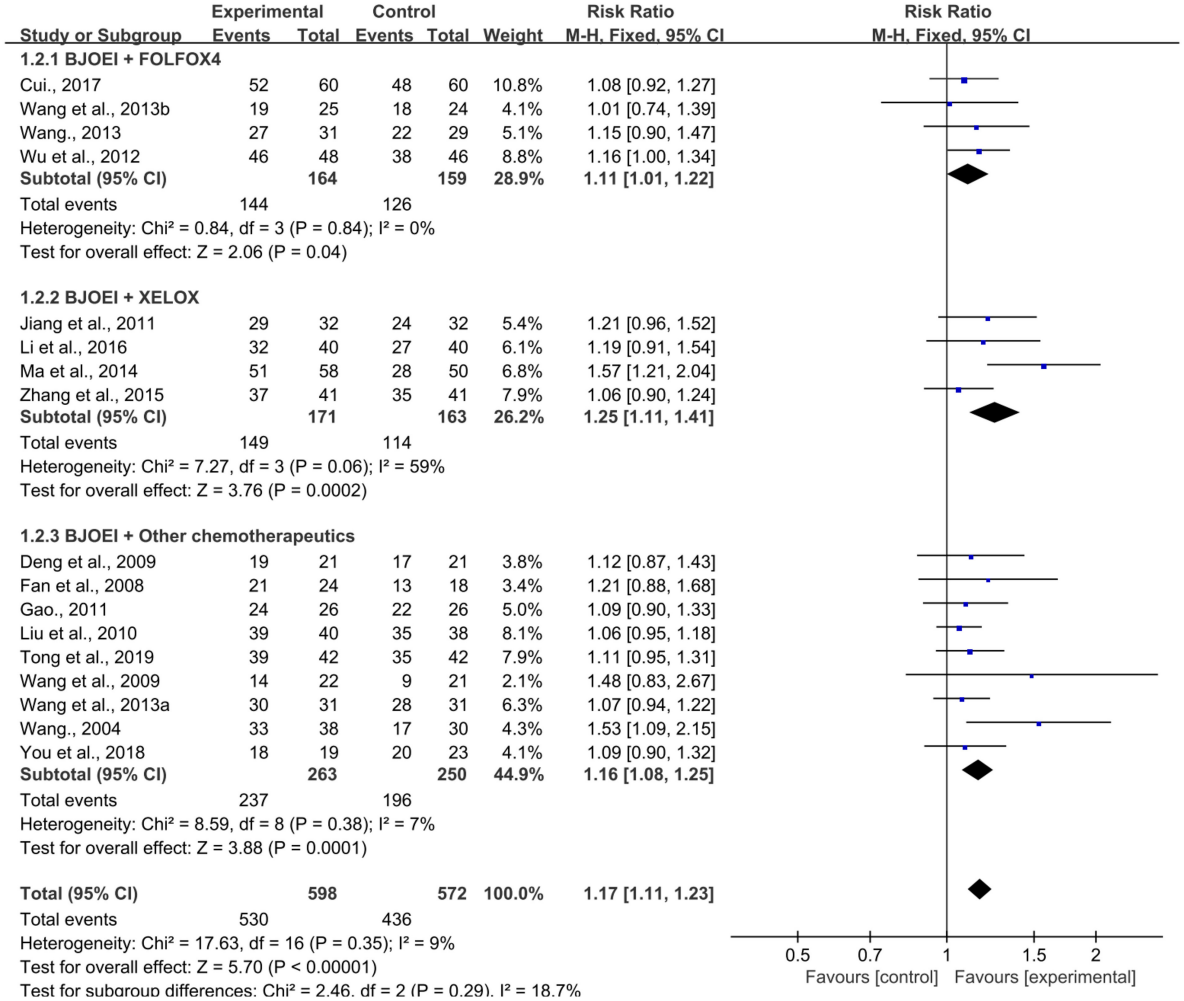
Forest plot of improvement of clinical benefit rate.

In total, 17 RCTs (10, 20, 22, 23, 25–33, 35–38) recorded CBR data. We conducted a subgroup analysis according to the different combination therapies of BJOEI, namely, BJOEI + FOLFOX4, BJOEI + XELOX, and BJOEI + other chemotherapeutics. As shown in Figure 4, the results demonstrated that, compared with the control group, the experimental group of patients with GC exhibited significantly improved CBR (RR = 1.17, 95% CI: 1.11–1.23, Z = 5.70, *P* < 0.00001). Subgroup analysis showed that there were statistically significant differences in CBR between the BJOEI intervention and control groups in patients who received BJOEI combined with FOLFOX4 (RR = 1.11, 95% CI: 1.01–1.22, Z = 2.06, *P* = 0.04), XELOX (RR = 1.25, 95% CI: 1.11–1.41, Z = 3.76, *P* = 0.0002), and other chemotherapeutics (RR = 1.16, 95% CI: 1.08–1.25, Z = 3.88, *P* = 0.0001).

#### Secondary Outcomes

##### Performance Status

**Figure 5:**
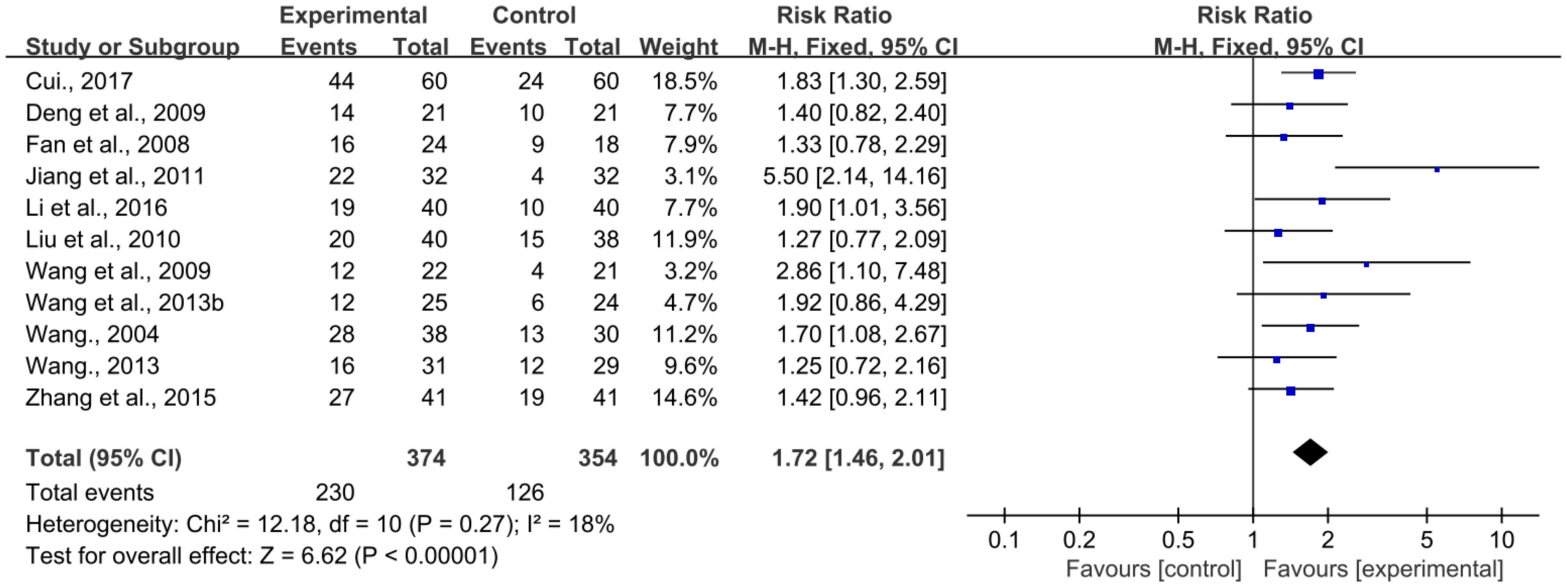
Forest plot of improvement of performance status.

As shown in Figure 5, 11 RCTs (20, 25, 26, 28–30, 32, 34–36, 38) reported the performance status data of the BJOEI and control groups with a slight heterogeneity (*P* = 0.27, *I*^2^ = 18% <50%). A meta-analysis demonstrated that the BJOEI group experienced ~72% superiority in terms of this outcome compared with the control group, and the difference was statistically significant (RR = 1.72, 95% CI: 1.46–2.01, Z = 6.62, *P* < 0.00001).

#### ADRs

**Figure 6:**
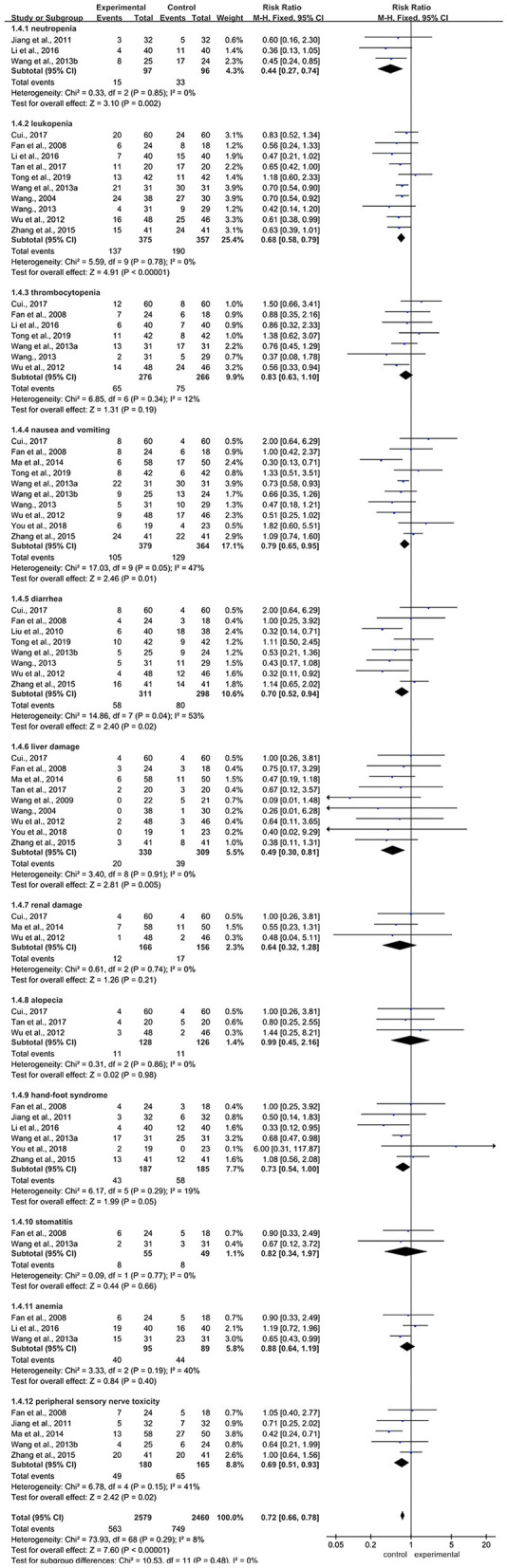
Forest plot of ADRs. ADRs, adverse drug reactions.

Sixteen RCTs referred to this outcome. The main ADRs were neutropenia (3 RCTs) (28, 29, 34), leukopenia (10 RCTs) (26, 29, 33, 35–38), thrombocytopenia (7 RCTs) (20, 22, 26, 29, 33, 36, 37), nausea and vomiting (10 RCTs) (20, 22, 23, 26, 31, 33, 34, 36–38), diarrhea (8 RCTs) (20, 22, 26, 30, 34, 36–38), liver damage (9 RCTs) (20, 21, 23, 26, 31, 32, 35, 37, 38), renal damage (3 RCTs) (20, 31, 37), alopecia (3 RCTs) (20, 21, 37), hand-foot syndrome (6 RCTs) (23, 26, 28, 29, 33, 38), stomatitis (2 RCTs) (26, 33), anemia (3 RCTs) (26, 29, 33), and peripheral sensory nerve toxicity (5 RCTs) (26, 28, 31, 34, 38). Meta-analysis showed that there was a statistically significant difference between the two groups (RR = 0.72, 95% CI: 0.66–0.78, Z = 7.60, *P* < 0.00001). Compared with the control group, the BJOEI group exhibited fewer of the following ADRs: neutropenia (RR = 0.44, 95% CI: 0.27–0.74, Z = 3.10, *P* = 0.002), leukopenia (RR = 0.68, 95% CI: 0.58–0.79, Z = 4.91, *P* < 0.00001), nausea and vomiting (RR = 0.79, 95% CI: 0.65–0.95, *Z* = 2.46, *P* = 0.01), diarrhea (RR = 0.70, 95% CI: 0.52–0.94, *Z* = 2.40, *P* = 0.02), liver damage (RR = 0.49, 95% CI: 0.30–0.81, *Z* = 2.81, *P* =0.005), hand-foot syndrome (RR = 0.73, 95% CI: 0.54–1.00, *Z* = 1.99, *P* = 0.05), and peripheral sensory nerve toxicity (RR = 0.69, 95% CI: 0.51–0.93, *Z* = 2.42, *P* = 0.02). However, no statistically significant differences were detected in the occurrence of thrombocytopenia, renal damage, alopecia, stomatitis, and anemia. The results of ADR were shown in Figure 6.

### Sensitivity Analysis

**Figure 7:**
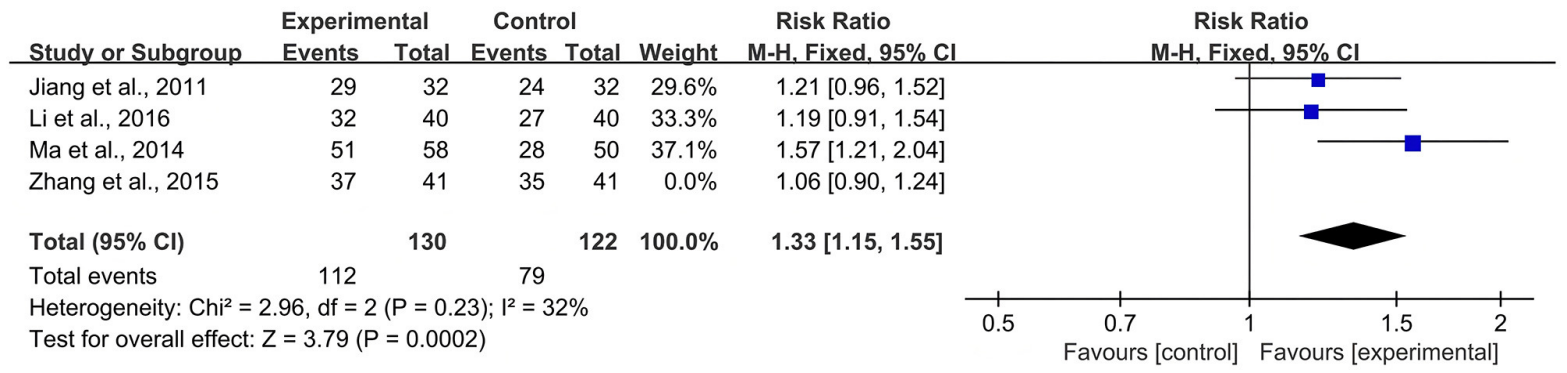
Forest plot of sensitivity analysis of CBR with BJOEI combined with XELOX treatment vs. pure XELOX treatment (a). CBR, clinical benefit rate; BJOEI, *Brucea javanica* oil emulsion injection.

**Figure 8:**
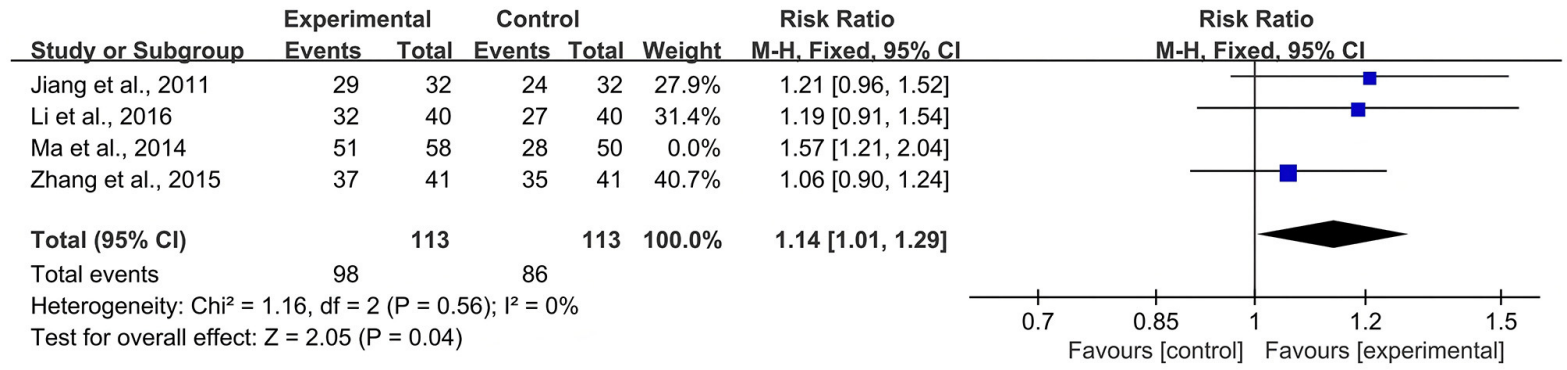
Forest plot of sensitivity analysis of CBR with BJOEI combined with XELOX treatment vs. pure XELOX treatment (b). CBR, clinical benefit rate; BJOEI, *Brucea javanica* oil emulsion injection.

**Figure 9:**
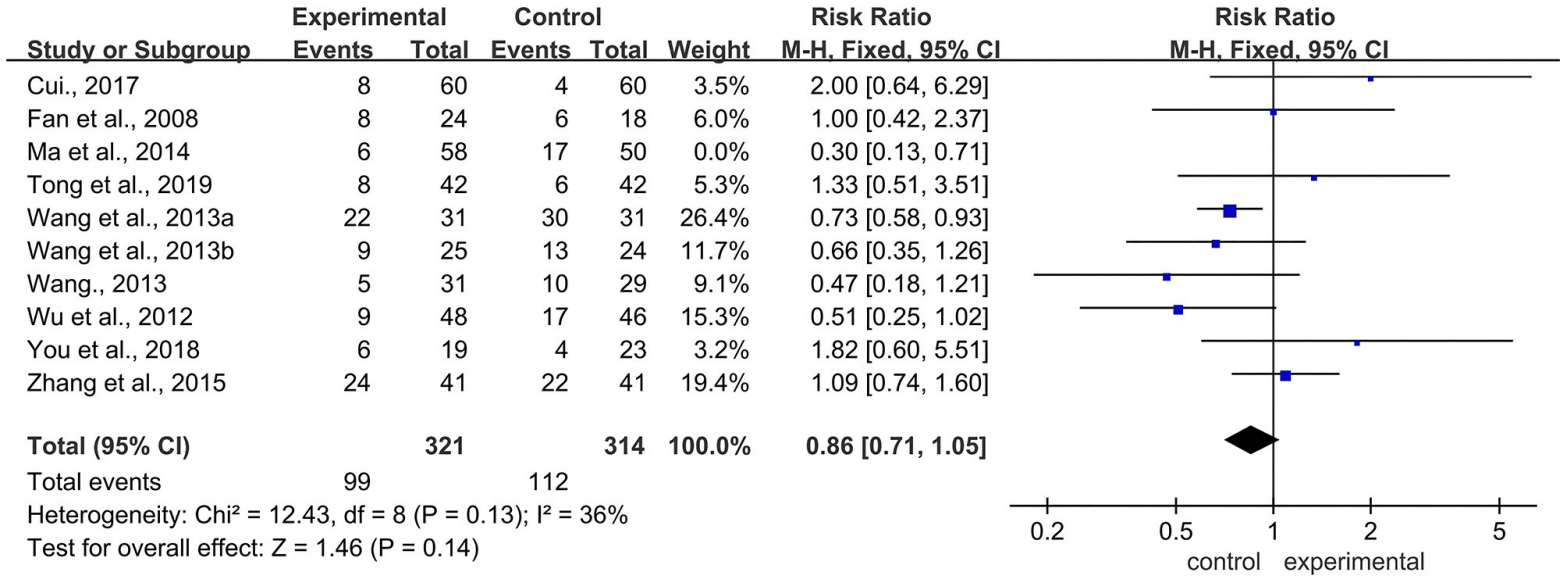
Forest plot of sensitivity analysis of ADRs of nausea and vomiting. ADRs, adverse drug reactions.

**Figure 10:**
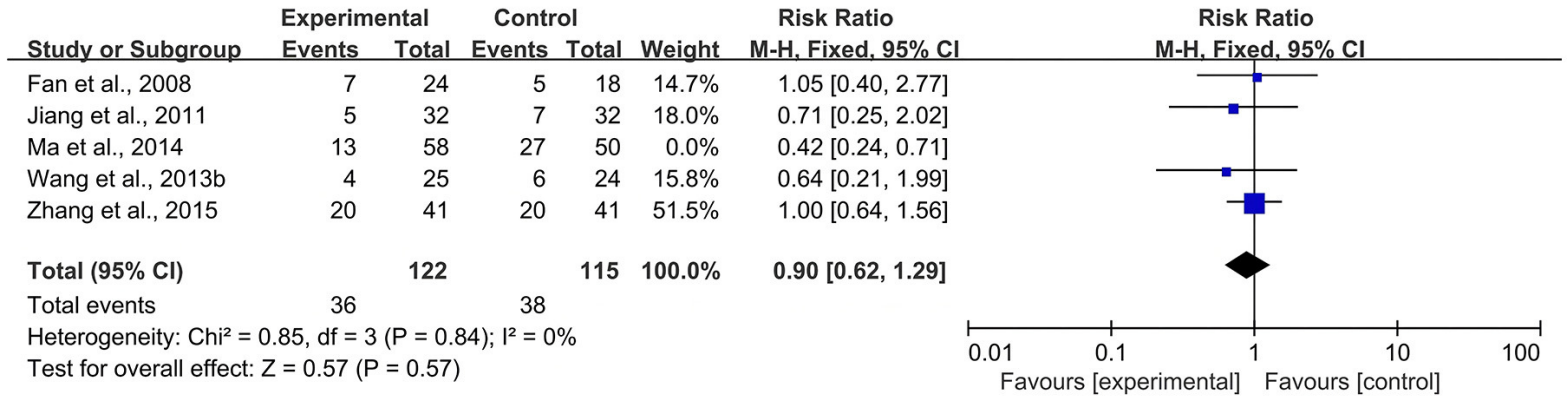
Forest plot of sensitivity analysis of ADRs of peripheral sensory nerve toxicity (a), ADRs, adverse drug reactions.

**Figure 11:**
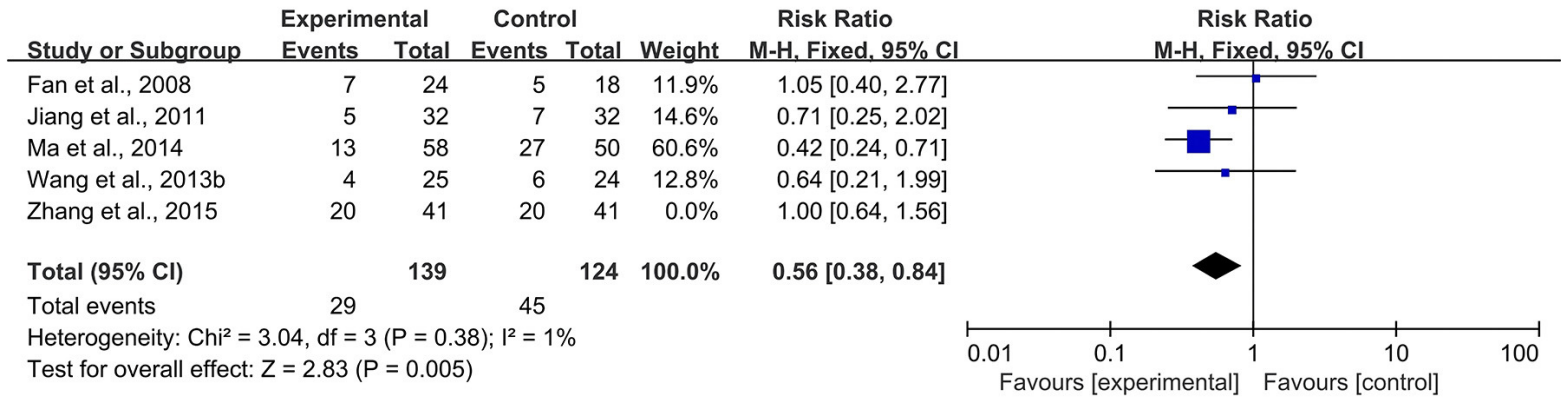
Forest plot of sensitivity analysis of ADRs of peripheral sensory nerve toxicity (b). ADRs, adverse drug reactions.

According to the *Cochrane Handbook for Systematic Reviews of Interventions* (39), *I*^2^ values between 0 and 40% indicated that heterogeneity might not be important. Therefore, we eliminated the included studies with *I*^2^ ≥ 40% one by one and then conducted a meta-analysis. The results showed that in the CBR of BJOEI + XELOX, after excluding Zhang et al. (38), the heterogeneity was decreased from 59 to 32% (*P* = 0.0002; Figure 7). After excluding Ma et al. (31), the heterogeneity was decreased from 59 to 0% (*P* = 0.04; Figure 8). The data suggested that Zhang et al. (38) and Ma et al. (31) were the main reasons for the heterogeneity in the CBR of BJOEI + XELOX. In terms of ADRs, after excluding Ma et al. (31), the heterogeneity of nausea and vomiting decreased was from 47 to 36% (*P* = 0.14; Figure 9), and the heterogeneity of peripheral sensory nerve toxicity was decreased from 41 to 0% (*P* = 0.57; Figure 10). In addition, after deleting Zhang et al. (38), the heterogeneity of peripheral sensory nerve toxicity was decreased from 41% to 1% (*P* = 0.005; Figure 11). These findings suggest that Zhang et al. (38) and Ma et al. (31) might explain the heterogeneity in ORR and ADRs.

### Analysis of Publication Bias

**Figure 12:**
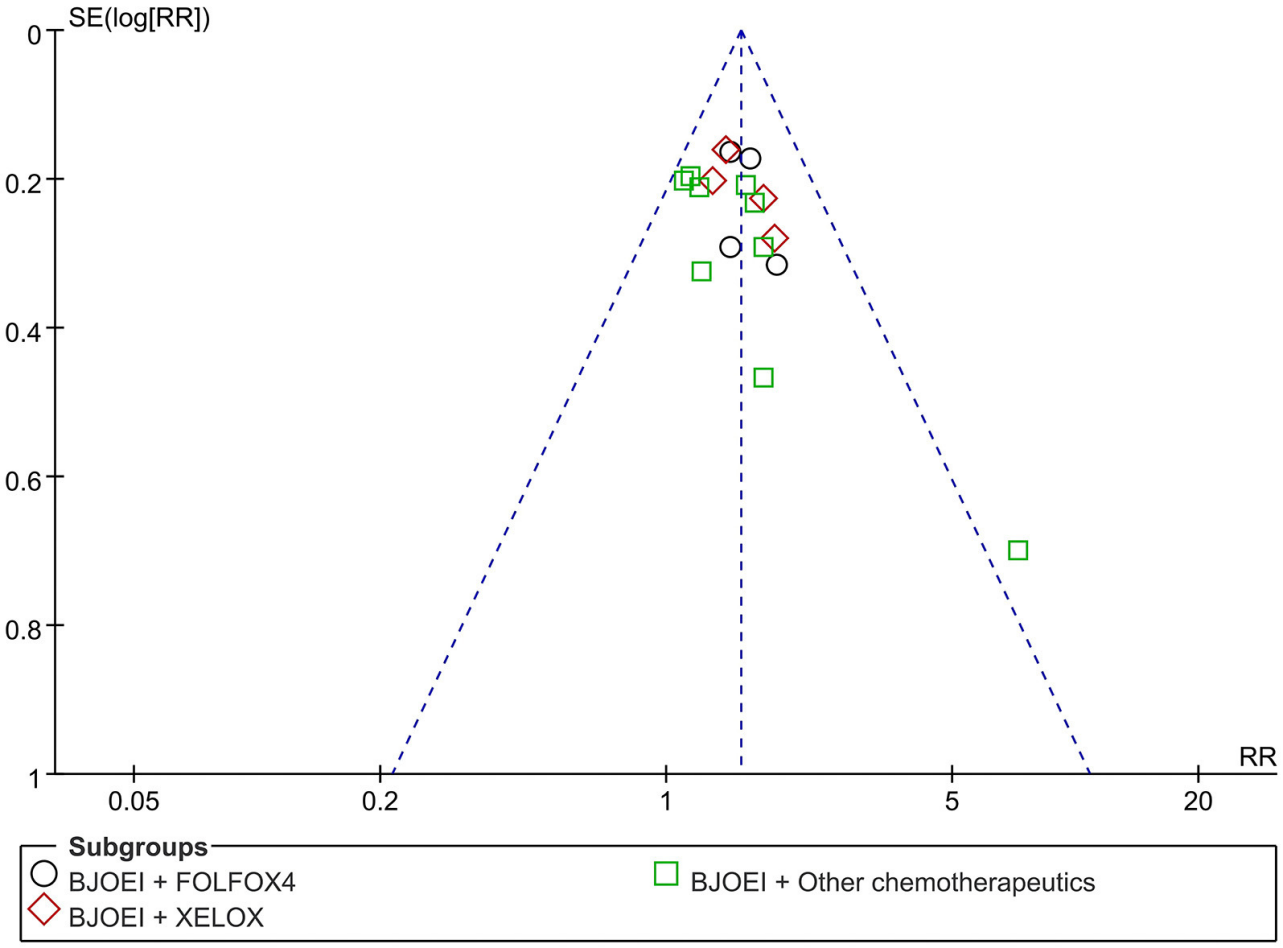
Funnel plot of ORR.

A funnel plot of publication bias for ORR is displayed in Figure 12, which indicates that there was no evidence of significant publication bias.

### Quality of Evidence Assessment

Based on the GRADE criteria, the ORR, CBR, performance status, and ADRs were all assessed as low-quality evidence, owing to the existence of clinical heterogeneity and low participant numbers in most studies (Tables 2, 3).

**Table 2:** Quality of evidence of primary outcomes.

***Brucea javanica*** **oil emulsion injection plus chemotherapy compared to chemotherapy for gastric cancer**					
**Patient or population**: **gastric cancer** **Setting**: **Randomized trials** **Intervention**: **Brucea javanica Oil Emulsion Injection plus chemotherapy** **Comparison: chemotherapy**					
**Outcome No of participants (studies)**	**Relative effect (95% CI)**	**Anticipated absolute effects (95% CI)**			**Certainty**
		**Risk without BJOEI**	**Risk with BJOEI**	**Difference**	
Overall response rate No of participants: 1170 (17 RCTs)	RR 1.52 (1.36–1.69)	42.5%	64.6% (57.8–71.8)	22.1% more (15.3 more to 29.3 more)	⊕⊕◯◯ LOW^a, b^
Overall response rate - FOLFOX4 No of participants: 323 (4 RCTs)	RR 1.55 (1.26–1.90)	43.4%	67.3% (54.7–82.5)	23.9% more (11.3 more to 39.1 more)	⊕⊕◯◯ LOW ^a, c^
Overall response rate - XELOX No of participants: 334 (4 RCTs)	RR 1.53 (1.24–1.88)	42.3%	64.8% (52.5–79.6)	22.4% more (10.2 more to 37.3 more)	⊕⊕◯◯ LOW ^a, c^
Overall response rate - Other chemotherapeutics No of participants: 513 (9 RCTs)	RR 1.48 (1.25–1.76)	42.0%	62.2% (52.5–73.9)	20.2% more (10.5 more to 31.9 more)	⊕◯◯◯ VERY LOW ^a, b, c^
Clinical benefit rate No of participants: 1170 (17 RCTs)	RR 1.17 (1.11–1.23)	76.2%	89.2% (84.6–93.8)	13.0% more (8.4 more to 17.5 more)	⊕⊕◯◯ LOW ^a, b^
Clinical benefit rate - FOLFOX4 No of participants: 323 (4 RCTs)	RR 1.11 (1.01–1.22)	79.2%	88.0% (80–96.7)	8.7% more (0.8 more to 17.4 more)	⊕⊕◯◯ LOW ^a, c^
Clinical benefit rate - XELOX No of participants: 334 (4 RCTs)	RR 1.25 (1.11–1.41)	69.9%	87.4% (77.6–98.6)	17.5% more (7.7 more to 28.7 more)	⊕◯◯◯ VERY LOW ^a, c, d^
Clinical benefit rate - Other chemotherapeutics No of participants: 513 (9 RCTs)	RR 1.16 (1.08–1.25)	78.4%	90.9% (84.7–98)	12.5% more (6.3 more to 19.6 more)	⊕◯◯◯ VERY LOW ^a, b, c^

^*^The risk in the intervention group (and its 95% CI) is based on the assumed risk in the comparison group and the relative effect of the intervention (and its 95% CI).

*RR, Risk ratio; BJOEI, Brucea javanica oil emulsion injection;*

a *Most information is from studies at unclear risk of bias;*

b *Clinical heterogeneity exists due to the different chemotherapy;*

c *Small sample size;*

d *Statistical heterogeneity exists*.

**Table 3:** Quality of evidence of secondary outcomes.

***Brucea javanica*** **oil emulsion injection plus chemotherapy compared to chemotherapy for gastric cancer**					
**Patient or population: gastric cancer** **Setting: Randomized trials** **Intervention:** ***Brucea javanica*** **Oil Emulsion Injection plus chemotherapy** **Comparison: chemotherapy**					
**Outcome** **No of participants (studies)**	**Relative effect** **(95% CI)**	**Anticipated absolute effects (95% CI)**			**Certainty**
		**Risk without BJOEI**	**Risk with BJOEI**	**Difference**	
performance status No of participants: 728 (11 RCTs)	RR 1.72 (1.46–2.01)	35.6%	61.2% (52–71.5)	25.6% more (16.4 more to 35.9 more)	⊕⊕◯◯ LOW ^a, b^
ADRs No of participants: 5039 (16 RCTs)	RR 0.72 (0.66–0.78)	30.4%	21.9% (20.1–23.7)	8.5% fewer (10.4 fewer to 6.7 fewer)	⊕⊕◯◯ LOW ^a, b^
ADRs - neutropenia No of participants: 193 (3 RCTs)	RR 0.44 (0.27–0.74)	34.4%	15.1% (9.3–25.4)	19.3% fewer (25.1 fewer to 8.9 fewer)	⊕◯◯◯ VERY LOW ^a, b, c^
ADRs - leukopenia No of participants: 732 (10 RCTs)	RR 0.68 (0.58–0.79)	53.2%	36.2% (30.9–42)	17.0% fewer (22.4 fewer to 11.2 fewer)	⊕⊕◯◯ LOW ^a, b^
ADRs - thrombocytopenia No of participants: 542 (7 RCTs)	RR 0.83 (0.63–1.10)	28.2%	23.4% (17.8–31)	4.8% fewer (10.4 fewer to 2.8 more)	⊕⊕◯◯ LOW ^a, b^
ADRs - nausea and vomiting No of participants: 743 (10 RCTs)	RR 0.79 (0.65–0.95)	35.4%	28.0% (23–33.7)	7.4% fewer (12.4 fewer to 1.8 fewer)	⊕⊕◯◯ LOW ^a, b^
ADRs - diarrhea No of participants: 609 (8 RCTs)	RR 0.70 (0.52–0.94)	26.8%	18.8% (14–25.2)	8.1% fewer (12.9 fewer to 1.6 fewer)	⊕◯◯◯ VERY LOW ^a, b, d^
ADRs - liver damage No of participants: 639 (9 RCTs)	RR 0.49 (0.30–0.81)	12.6%	6.2% (3.8–10.2)	6.4% fewer (8.8 fewer to 2.4 fewer)	⊕⊕◯◯ LOW ^a, b^
ADRs - renal damage No of participants: 322 (3 RCTs)	RR 0.64 (0.32–1.28)	10.9%	7.0% (3.5–13.9)	3.9% fewer (7.4 fewer to 3.1 more)	⊕⊕◯◯ VERY LOW ^a, b, c^
ADRs - alopecia No of participants: 254 (3 RCTs)	RR 0.99 (0.45–2.16)	8.7%	8.6% (3.9–18.9)	0.1% fewer (4.8 fewer to 10.1 more)	⊕◯◯◯ VERY LOW ^a, b, c^
ADRs - hand-foot syndrome No of participants: 372 (6 studies)	RR 0.73 (0.54–1.00)	31.4%	22.9% (16.9–31.4)	8.5% fewer (14.4 fewer to 0 fewer)	⊕◯◯◯ VERY LOW ^a, b, c^
ADRs - stomatitis No of participants: 104 (2 RCTs)	RR 0.82 (0.34–1.97)	16.3%	13.4% (5.6–32.2)	2.9% fewer (10.8 fewer to 15.8 more)	⊕◯◯◯ VERY LOW ^a, b, c^
ADRs - anemia No of participants: 184 (3 RCTs)	RR 0.88 (0.64–1.19)	49.4%	43.5% (31.6–58.8)	5.9% fewer (17.8 fewer to 9.4 more)	⊕◯◯◯ VERY LOW ^a, b, c^
ADRs - peripheral sensory nerve toxicity No of participants: 345 (5 RCTs)	RR 0.69 (0.51–0.93)	39.4%	27.2% (20.1–36.6)	12.2% fewer (19.3 fewer to 2.8 fewer)	⊕◯◯◯ VERY LOW ^a, b, c^

^*^The risk in the intervention group (and its 95% CI) is based on the assumed risk in the comparison group and the relative effect of the intervention (and its 95% CI).

*RR, Risk ratio; BJOEI, Brucea javanica oil emulsion injection;*

a *Most information is from studies at unclear risk of bias;*

b *Clinical heterogeneity exists due to the different chemotherapy;*

c *Small sample size;*

d *Statistical heterogeneity exists*.

## Discussion

Despite advances in disease screening and modern technology, GC remains one of the most common malignant tumors. Its metastasis, morbidity, and mortality rates are all on the rise, while the cure, radical resection, and 5-year postoperative survival rates of patients with advanced GC are low (40). In recent years, TCM has made great progress in anti-tumor therapy, and the manufacturing technologies of Chinese medicine compounds, Chinese patent medicine, Chinese medicine extract, and Chinese medicine monomers have developed more rapidly. BJOEI is a Chinese patent medicine that is widely used in the treatment of various cancers, such as lung (41) and several gastrointestinal cancers (42, 43). Previous studies have shown that its antitumor effects might be related to the following mechanisms: 1) inhibition of DNA synthesis in tumor cells (44, 45); 2) induction of tumor cell apoptosis and differentiation (46–48); 3) anti-angiogenesis (49); and 4) reversion of drug resistance (50).

In this study, we searched as many RCTs as we could and conducted a meta-analysis to evaluate the treatment efficacy and safety of BJOEI in patients with GC. All available data from the collected trials were applied without intentional selection. The results showed that BJOEI combined with chemotherapy was superior to single chemotherapy in improving ORR, CBR, and performance status. Considering that the different patient regimens might lead to high outcome heterogeneity, to obtain a more convincing conclusion, we conducted a subgroup analysis according to chemotherapeutic regimens. The results showed that for each BJOEI + FOLFOX4 and BJOEI + XELOX sub-group, the ORR and CBR were significantly improved by the addition of the BJOEI intervention. Furthermore, we have paid special attention to neutropenia, leukopenia, nausea and vomiting, diarrhea, liver damage, hand-foot syndrome, and peripheral sensory nerve toxicity, which are common symptoms of chemotherapy-associated ADRs. The meta-analysis showed that the BJOEI group had fewer symptoms related to the above ADRs. However, more RCTs are needed to further demonstrate the positive effect of BJOEI in ameliorating chemotherapy-associated toxicities.

Although we strictly conducted this meta-analysis according to the review procedure released by the Cochrane Collaboration, this study has several limitations. First, the duration of the intervention is an important factor in the evaluation of efficacy. The observation time of the included studies was mainly concentrated at 12 and 6 W, and the longest was 24 W (in only one RCT). Furthermore, the long-term effects of BJOEI in the treatment of GC remain unknown. Moreover, high-quality original studies were scarce in this study. The problems in most RCTs included unexplained randomization methods, insufficient attention to allocation concealment, low utilization rate of blinding, and unreported lost follow-up cases. Finally, recent advances have renewed the hope that immune and targeted agents can be leveraged to improve patient survival (51, 52). Although chemotherapy is still the backbone of therapy against GC, studies should also investigate the efficacy of BJOEI combined with immunotherapy or targeted therapy.

Due to the limitations associated with the poor quality of pooled studies, it is difficult to draw a definitive conclusion. Nevertheless, our study suggests the positive effect of BJOEI in facilitating the management of ORR, CBR, performance status, and ADRs in patients with GC. More prospectively designed, large-sample, and multicenter RCTs are expected to offer persuasive evidence to demonstrate the efficacy and safety of BJOEI.

## Author Contributions

JL designed this study. XW and HW performed the online database search. LC, JW, TL, and SL contributed to the data collection, extraction, and analysis. XW, HW, and LC prepared the original draft and finished the revision of the manuscript. All authors have read and approved the final manuscript.

## Conflict of Interest

The authors declare that the research was conducted in the absence of any commercial or financial relationships that could be construed as a potential conflict of interest.

## Publisher's Note

All claims expressed in this article are solely those of the authors and do not necessarily represent those of their affiliated organizations, or those of the publisher, the editors and the reviewers. Any product that may be evaluated in this article, or claim that may be made by its manufacturer, is not guaranteed or endorsed by the publisher.
